# How astrocyte networks may contribute to cerebral metabolite clearance

**DOI:** 10.1038/srep15024

**Published:** 2015-10-14

**Authors:** Mahdi Asgari, Diane de Zélicourt, Vartan Kurtcuoglu

**Affiliations:** 1 The Interface Group, Institute of Physiology, University of Zurich, Zurich, Switzerland; 2 Neuroscience Center Zurich, University of Zurich, Zurich, Switzerland; 3 Zurich Center for Integrative Human Physiology, University of Zurich, Zurich, Switzerland

## Abstract

The brain possesses an intricate network of interconnected fluid pathways that are vital to the maintenance of its homeostasis. With diffusion being the main mode of solute transport in cerebral tissue, it is not clear how bulk flow through these pathways is involved in the removal of metabolites. In this computational study, we show that networks of astrocytes may contribute to the passage of solutes between tissue and paravascular spaces (PVS) by serving as low resistance pathways to bulk water flow. The astrocyte networks are connected through aquaporin-4 (AQP4) water channels with a parallel, extracellular route carrying metabolites. Inhibition of the intracellular route by deletion of AQP4 causes a reduction of bulk flow between tissue and PVS, leading to reduced metabolite clearance into the venous PVS or, as observed in animal studies, a reduction of tracer influx from arterial PVS into the brain tissue.

Homeostasis of the central nervous system is critically dependent on cerebrospinal and interstitial fluid flows and therewith associated transport processes. While it is possible to measure cerebrospinal fluid (CSF) flow *in vivo* at some locations^1^, flow of the interstitial fluid (ISF) cannot be acquired directly, but only be inferred from the evolution of tracer distribution patterns^2^,^3^,^4^. However, tracers can spread in absence of flow simply driven by diffusion^5^, and they can also spread in the absence of both diffusion and net flow by local advective mixing^6^. Consequently, a given tracer distribution in the brain could be the result of one of several possible flow fields. The interpretation of tracer studies with respect to the underlying flow field is thus anything but trivial.

Recent *in vivo* two-photon excitation microscopy studies in mice have addressed the role of aquaporin-4 (AQP4) water channels on the transport of fluorescent tracers in the brain^7^. In mice lacking AQP4, the time for tracers injected into the CSF to reach the ISF through paravascular spaces (PVS) was increased, which was interpreted as a consequence of reduced CSF influx into the PVS. While this interpretation appears intuitive at first, it becomes much less evident on second thought: Both fluid and tracers pass from the PVS to the ISF via gaps between astrocyte endfeet, but only fluid leaves the PVS additionally through AQP4. If this second pathway is removed, there should not be reduced fluid passage through the first and the time for tracers to reach the ISF should not increase.

Light may be shed onto this challenging constellation if one considers that AQP4 does not connect the PVS directly to the ISF, but rather connects it to the intracellular space of astrocytes through their endfeet first^8^. The intracellular space then communicates with the ISF via AQP4 expressed on the entire plasma membrane. Since astrocytes can exchange water both through the surrounding ISF and directly through gap junction connections with neighbouring astrocytes, fluid originating in the arterial PVS may flow through a network of astrocytes before reaching the venous PVS^9^,^10^. As tracers will not pass through AQP4, and tracking of labelled water cannot be achieved *in vivo* with sufficiently high resolution, a computational approach to the analysis of water flow between paravascular spaces is warranted. Computational methods for the study of cerebral fluid dynamics have made great strides in recent years, and have become valuable tools that can complement experimental approaches^11^,^12^,^13^,^14^.

Herein we aim to elucidate through numerical experiments the changes in fluid flow that lead to altered tracer distribution patterns upon AQP4 deletion observed *in vivo*. The underlying mathematical model considers both intra- and extracellular water pathways in a network of astrocytes between two neighbouring arterial and venous PVS. Based on the calculated fluid flow, we further comment on the relative contributions of advection and diffusion to solute transport in the extracellular and paravascular spaces.

## Methods

**Figure 1:**
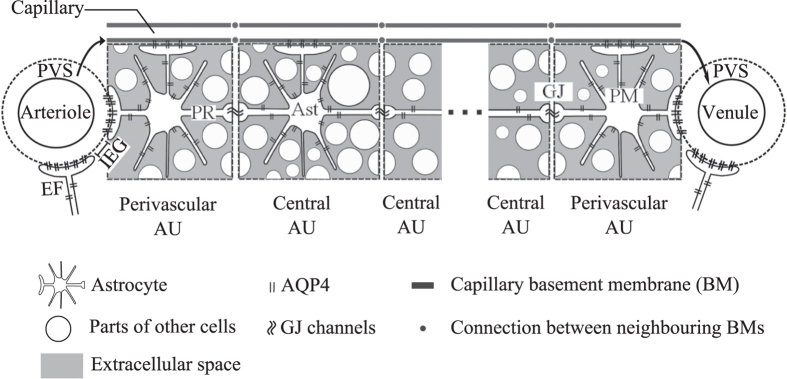
Sketch of the model domain consisting of an astrocyte network between arterial and venous paravascular spaces (PVS). The network consists of several astrocyte units (AU) that each include one astrocyte (Ast) expressing aquaporin-4 (AQP4) water channels, extracellular space (ECS) and parts of the intracellular space of other cells including neurons, oligodendrocytes and microglia. Note that the schematic is not drawn to scale and does not represent the true ratio between intra- and extracellular space. The perivascular AUs are in direct contact with their neighbouring PVS via the respective astrocyte’s endfoot (EF). All astrocytes connect via endfeet to the basement membrane (BM) of the capillary associated with their AU. Neighbouring BMs connect to each other, and the perivascular BMs are additionally connected to the corresponding PVS (indicated by arrows in the figure). AQP4 covering each of these endfeet connect the capillary BMs or the PVS to the intra-astrocyte space. The intra-astrocyte and extracellular spaces within a given AU are in constant exchange of water through AQP4 water channels expressed in the astrocyte plasma membrane (PM). Gap junctions (GJ) connect the intracellular spaces of two adjacent astrocytes. PR: Astrocyte process. IEG: Inter-endfeet-gap.

The model represents the space between a penetrating cortical arteriole and its venous counterpart in a mouse brain as shown in Fig. 1. The vessels are spaced 300 μm apart^10^. *In vivo*, astrocytes form a structured network between adjacent larger blood vessels^15^. Each astrocyte can be thought of possessing its own domain with nominal diameter of 50 μm^10^. We refer to this domain as astrocyte unit (AU). An AU contains one astrocyte, parts of other cerebral cell types such as neurons, oligodendrocytes and microglia, as well as extracellular space at an overall ratio of 1:4 between extra- and intracellular spaces. Each AU is associated with one capillary blood vessel (see Supplementary Information S1).

There are nominally six AUs between arteriole-venule pairs, where one AU is in direct contact with the arterial and one with the venous PVS. These two units are referred to as perivascular AUs, while the remaining four constitute the central astrocyte units. All AUs are in contact with the basement membrane (BM) of their own associated capillary^16^. Capillary basement membranes of neighbouring AUs are in direct contact, representing the connectivity of the capillary bed. The capillary basement membranes of the two perivascular AUs connect additionally to their respective adjacent PVS.

Astrocyte endfeet establish the contact between AU and PVS, as well as between AU and capillary basement membrane^17^, and thereby define two parallel fluid pathways: One through AQP4 expressed on the endfoot plasma membrane, and one through gaps between adjacent endfeet. Such inter-endfeet gaps (IEG) also allow the passage of solutes with hydrodynamic diameter of up to 20 nm^7^,^17^, whereas AQP4 only carries water. In the central part of the network, intra- and extracellular spaces and capillary basement membranes act as parallel fluid pathways that are in a constant exchange of water through AQP4 expressed on the astrocyte plasma membrane. The intracellular spaces of neighbouring astrocytes can further communicate directly through gap junction proteins, which are fairly large channels with hydrodynamic diameter of 2.5–4.5 nm^18^ that allow for passage of molecules with molecular mass less than 1000 Da^10^.

*In vivo* tracer studies suggest the existence of net flow from the arterial to venous PVS, and therefore of a driving force gradient between the two spaces^7^. Neither origin nor magnitude of this purported gradient is known: next to arterial wall pulsations that are hypothesized to produce a driving hydrostatic pressure gradient, other driving forces are plausible as well. To reproduce the net effect of several possible forces and to employ a reasonable gradient magnitude, we set a pressure difference between arterial and venous PVS that yields a baseline extracellular fluid flow velocity in line with experimentally estimated values in grey matter^19^. As we will discuss in Supplementary Information S3, variation of the gradient magnitude within a reasonable range does not affect the conclusions of our investigation.

**Figure 2:**
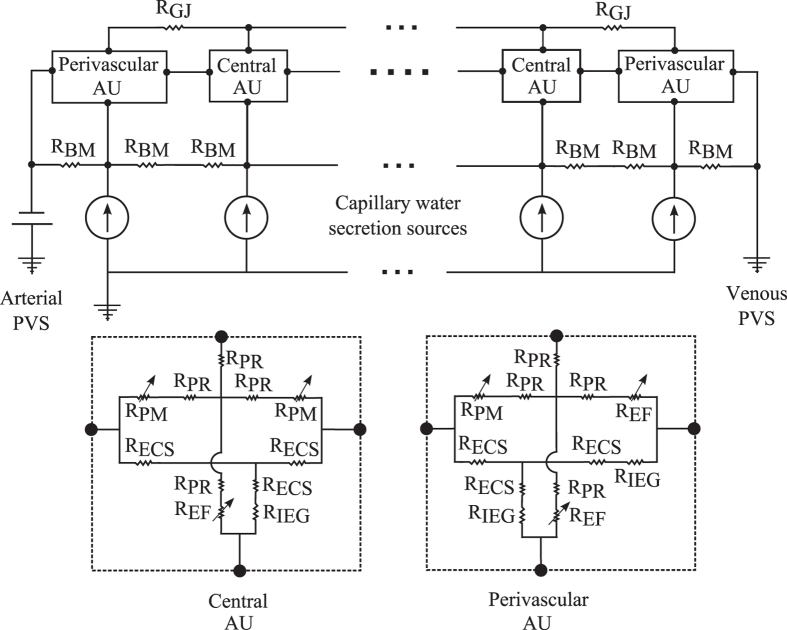
Electrical analogue model of cerebral water transport between arterial and venous paravascular spaces (PVS). Definitions of the abbreviations referring to the physical model domain are given in Fig. 1. The voltage source represents the driving pressure difference between arterial and venous paravascular spaces that are connected by resistances (R) representing the resistance to fluid flow of capillary basement membrane (BM) segments and astrocyte units (AU). Each astrocyte unit (AU) includes resistances of both intracellular (cell processes, PR) and extracellular (ECS) pathways which are linked by membrane resistances, namely those of the astrocyte endfoot membrane (EF) and the remainder of the astrocyte plasma membrane (PM). Since these membrane resistances are dependent on the AQP4 expression level, they are indicated as variable resistances (arrows). Gap junction (GJ) resistances connect the intracellular spaces of two neighbouring astrocytes.

The computational implementation of the model is based on electrical analogy to fluid flow: pressure, resistance to flow and flow rate are represented by electric potential, resistance and current, respectively. Figure 2 shows the corresponding electrical network. The overall pressure drop is set to where under nominal conditions ISF flow velocity reaches 1 μm/min, corresponding to experimental estimates in the grey matter^19^. A sensitivity analysis reported in Supplementary Information S3 shows that changing the nominal ISF flow velocity within the range of values reported in the literature^20^ does not affect the conclusions drawn in this study.

The distribution of flow between pathways depends on the resistances along those pathways. These include a) the resistances within the intra- and extracellular spaces and capillary basement membranes determined by their respective characteristic dimensions and properties; b) the interfacial resistances between astrocyte intracellular space and ECS, PVS or BM, divided between endfoot and the remainder of the plasma membrane; and c) the interfacial resistance between neighbouring astrocytes determined by the gap junction resistance to water passage. Values of the resistances and the driving pressure gradient are shown in Table 1 along with their derivation in concise form. Details are given in Supplementary Information S1. The underlying experimental values are listed in Table 2.

Next to water inflow from the arterial PVS, there is also the possibility of water secretion from capillaries into the parenchyma^4^. Since the experimental evidence for such secretion is unsatisfactory and no reliable quantitative data on the corresponding flow rate exist^21^, we exclude capillary water secretion in the main calculations, but investigate its possible effects with an expanded model shown in Supplementary Fig. S2 and described in detail in Supplementary Information S1. Briefly, water secretion from the capillaries into the AUs is enforced and the pressure gradient between arterial and venous PVS is reduced so that the nominal flow rate through the tissue (combination of influx from the arterial PVS and secretion by capillaries) is maintained. As in the main model, AQP4 deletion is taken into account through a sevenfold increase in the astrocyte membrane resistances and, in addition, through a 31% reduction in capillary secretion rate as observed in glial-conditional AQP4 knockout mice after systemic hypoosmotic stress^22^. This explicit reduction is necessary because the capillary water secretion rate rather than a driving pressure gradient is prescribed. As shown in the sensitivity analysis in Supplementary Information S3, variation of the capillary secretion rate reduction within reasonable bounds does not affect the conclusions drawn in this study as long as the arterial PVS is the main source of water flux into the parenchyma.

## Results

### Baseline flow rates and flow distribution between intra- and extracellular routes

**Figure 3:**
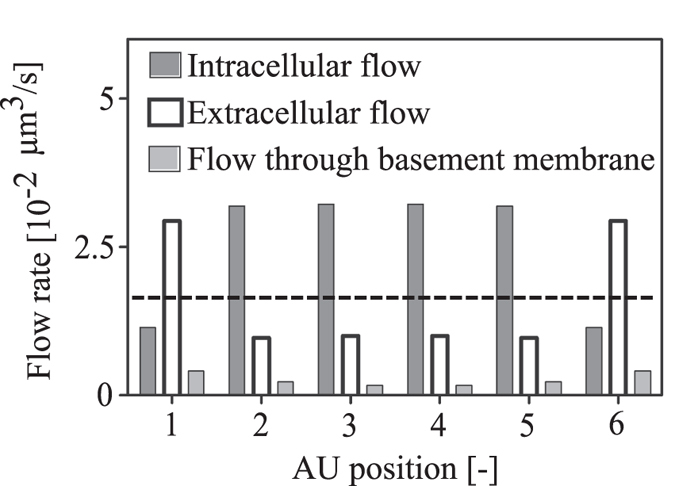
Flow rate distribution between intracellular, extracellular and capillary basement membrane pathways under normal conditions. AU Positions 1 and 6 refer the arterial and venous perivascular astrocyte units, respectively. The remaining positions refer to the central AUs. The dashed line indicates hypothetical extracellular flow rate (not including flow through basement membrane) in absence of the parallel, interconnected intracellular pathway.

To analyse the relative contributions of the intra- and extracellular fluid routes, we probed the model under baseline conditions with all pathways open and all resistances set to their nominal value as described in the Methods section. Water enters the brain tissue from the arterial PVS through gaps between astrocyte endfeet and through AQP4 expressed on astrocyte endfeet at a ratio of about 3:1. This is illustrated in Fig. 3 astrocyte unit (AU) position 1. Once inside the tissue, there is continuous exchange of water between the intra- and extracellular spaces through AQP4 channels covering the astrocyte cell body and processes. With the ECS and capillary basement membrane having a higher resistance to fluid flow than the intracellular space, water moves preferentially through the latter at a ratio of 3:1 (see Fig. 3, AU positions 2 to 5). Flow distribution at the exit into the venous PVS is similar to the one at entry from the arterial PVS (AU position 6). At these entry and exit positions, flow rate through the ECS is 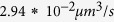, and reduces to
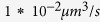
 deeper in the brain tissue, yielding a mean ECS flow rate of 
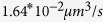
. In absence of the intracellular route, the flow rate through the ECS would reach a similar value of 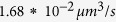 at all probed positions, but this would correspond to the overall flow rate from arterial to venous PVS (Fig. 3, dashed horizontal line) through the parenchyma and would be less than half of the total water flow rate achieved with the astrocytes present (intra- and extracellular flows combined, 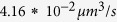).

### Dependence of flow rate on number of astrocytes between arterial and venous PVS

**Figure 4:**
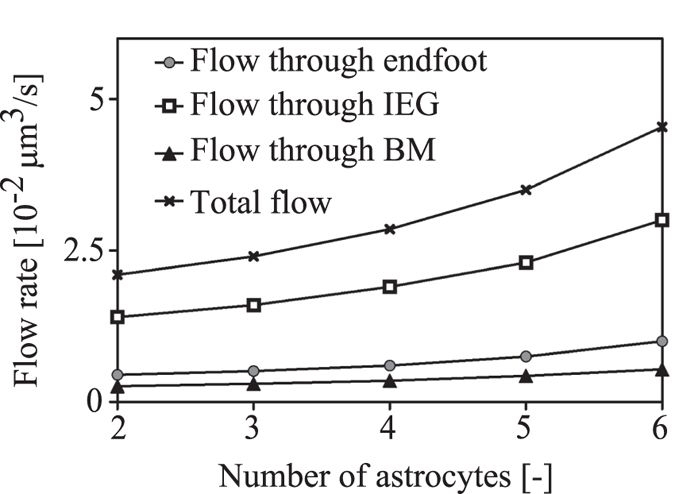
Water flow rate from arterial PVS into the parenchyma as a function of the number of astrocytes between adjacent PVS. 6 astrocytes in this space correspond to the baseline, whereas the lowest shown number of 2 only includes the perivascular astrocytes. In configurations with less than 6 astrocytes, the resulting gaps are assumed to be filled with generic cells that do not express AQP4.

To quantify the contribution of the central astrocytes to fluid flow, we gradually reduced the number of astrocytes between adjacent PVS from six (baseline) to two (only perivascular astrocytes). The resulting gaps were filled with generic cells that do not express AQP4. Since in the central nervous system AQP4 channels were shown to be predominantly expressed in astrocytes^23^,^24^, these generic cells can be neurons, oligodendrocytes, microglia or any cells of the central nervous system other than astrocytes. As illustrated in Fig. 4, lowering the number of central astrocytes leads to reduced flow between paravascular spaces, affecting water entry from the arterial PVS through IEG more than it affects entry through endfeet.

### Dependence of flow rate on AQP4 expression

**Figure 5:**
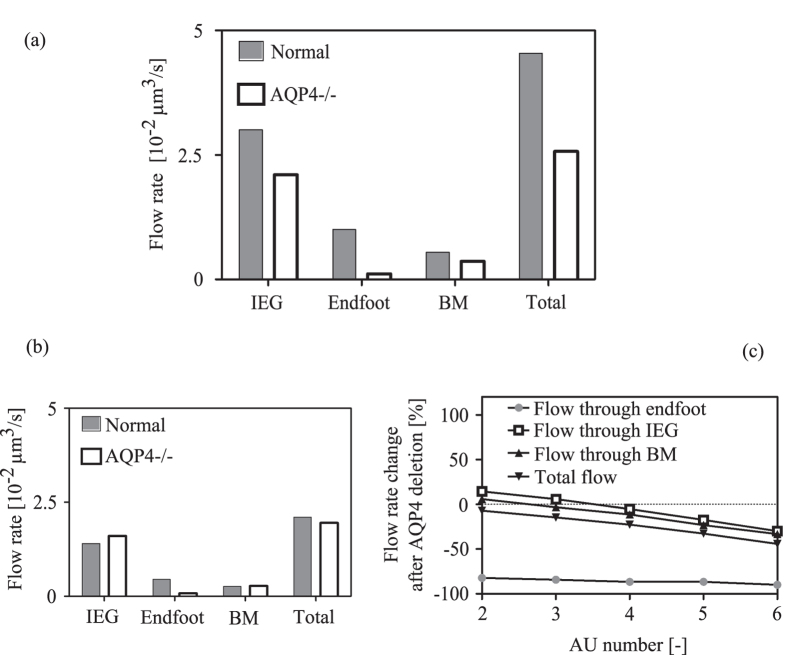
Effect of AQP4 deletion on water flow rate from PVS to the parenchyma through endfoot AQP4 channels, inter-endfeet-gaps (IEG), capillary basement membrane (BM) and in total. (**a**) Baseline configuration with 6 astrocytes. (**b**) Configuration with only the two perivascular astrocytes with the remaining space filled with other cells not expressing AQP4. (**c**) Percentage change in flow rates from PVS to tissue after AQP4 deletion as a function of the number of AUs included in the network.

To analyse the dependence of flow on AQP4, we disabled flow through these water channels, thereby replicating the experimental situation found in the *in vivo* tracer distribution study in AQP4 knock-out mice by Iliff and colleagues^7^. Deletion of AQP4 does not render the astrocyte membrane completely impermeable to water, but rather translates into a sevenfold reduction in overall water conduction of the astrocyte membrane^25^. We modelled this as a sevenfold increase in overall plasma and endfoot membrane resistances (see Supplementary Information S1). As one would expect, deletion of AQP4, which are polarized on the astrocyte endfeet, reduces water entry into the brain tissue through the endfeet substantially (Fig. 5a, 90% reduction). Far less intuitive, but in agreement with the mentioned experimental study^7^, AQP4 deletion also reduces flow through IEG markedly (Fig. 5a, 30% reduction) and flow through the capillary basement membrane by 33%. The combined effect is a 43.4% reduction in the total flow rate. When the number of astrocytes between arterial and venous PVS is reduced from the baseline number of six to two, AQP4 deletion leads to a 14% increase (rather than decrease) in flow through IEG (Fig. 5b) and a reduction of total flow rate by only 7%. The flow rate changes in response to AQP4 knock-out as a function of the astrocyte coverage is shown in Fig. 5c.

**Figure 6:**
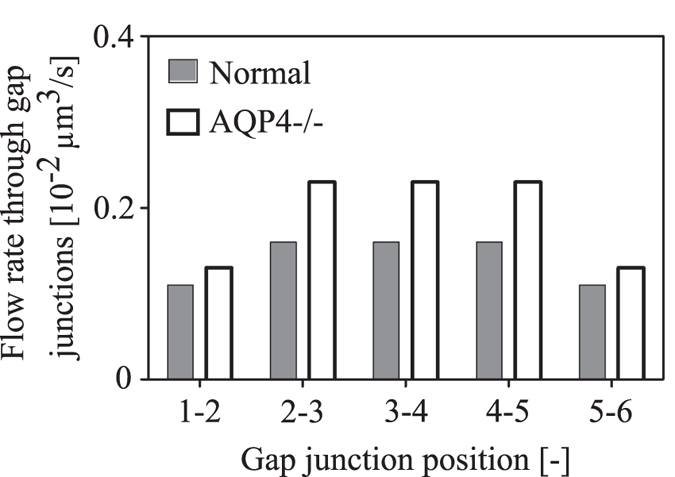
The effect of AQP4 deletion on water flow rate through the direct GJ connections between neighbouring astrocytes in the network. Gap junction positions 1–2 and 5–6 refer to the connection between the arterial perivascular astrocyte and the first central astrocyte, and between the last central astrocyte and venous perivascular astrocyte, respectively.

Deletion of AQP4 also affects flow through gap junctions, as shown in Fig. 6. Consistent with experimental observations of increased inter-cellular tracer spread in AQP4 knock-out^26^, flow through GJ is increased when AQP4 is deleted.

### Dependence of flow rate on AQP4 polarization

To assess the influence of AQP4 polarization on flow rate and flow distribution, we distributed AQP4 uniformly over the entire astrocyte plasma membrane. This is in contrast to the baseline situation in which AQP4 populates the endfoot with ten times higher density than it does the remainder of the plasma membrane^27^. AQP4 depolarization thus leads to a tenfold increase in resistance to water flow through the endfoot compared to baseline (from 
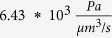
 to 
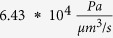
), while the resistance of the remaining membrane is only marginally reduced (from 
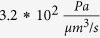
 to 
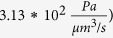
, owing to its large surface area compared to that of the endfoot.

**Figure 7:**
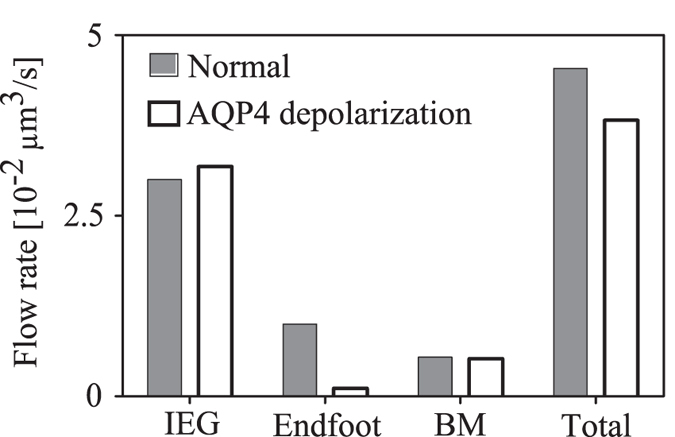
Effect of AQP4 depolarization on water flow rate from PVS to the parenchyma through endfoot AQP4 channels, inter-endfeet-gaps (IEG), capillary basement membrane (BM) and in total. Dark bars refer to the baseline state with AQP4 water channels polarized on the endfoot, whereas light bars refer to the depolarized state.

Depolarization of AQP4 causes an 89% reduction in water entry from the PVS through the endfoot (Fig. 7). This is accompanied by a 6% increase in inflow through IEG and a 3.7% reduction in flow through the capillary basement membrane. The total flow rate is reduced by 15.9%.

### Influence of capillary water secretion on flow rate

**Figure 8:**
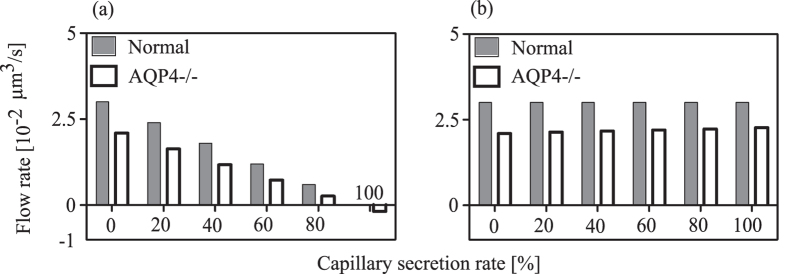
Effect of capillary water secretion rate on the water flow rate through inter-endfeet gaps connecting the perivascular AUs to the (a) arterial and (b) venous PVS, respectively. The left most pair of bars in both panels show values for zero water secretion, whereas the right most pairs bars give the values for the case where all of the water inflow into the domain stems from capillary secretion. The white bars represent the AQP4 deletion state, in which next to the removal of the corresponding pathway in the AUs, a reduction of the capillary water secretion by 31% is imposed^22^.

To test the influence of water secretion from capillaries on changes in flow rate through inter-endfeet gaps upon AQP4 deletion, we employed the expanded model shown in Supplementary Fig. S2. Having now two sources of inflow into the modelled space between arterial and venous PVS, we varied the contribution of capillary secretion to total inflow from 0 to 100% in six steps, recording the flow rate through perivascular inter-endfeet gaps (Fig. 8). Increasing the contribution of capillary secretion reduces inflow through IEG on the arterial side (Fig. 8a), but does not change outflow through perivenous IEG noticeably (Fig. 8b).

Just as in the model without water secretion by capillaries, AQP4 deletion leads to a reduction in flow rate through periarterial IEG, as long as the capillaries’ contribution does not exceed 80% of the overall inflow into the parenchyma. On the venous side, the capillary secretion rate influences the change in flow rate through IEG from baseline to AQP4 knock-out only slightly. It should be noted that the behavior of an incomplete astrocyte network as described in the section ‘The Dependence of flow rate on number of astrocytes between arterial and venous PVS’ is more sensitive to variations in capillary water secretion. This is discussed in Supplementary Information S3.

### Sensitivity analysis

To evaluate the sensitivity of the reported results on the choice of parameter values, we assessed the model output for parameter variations within realistic bounds. Results of the sensitivity analysis are reported in Supplementary Information S3. Consistent behaviours were noted in all cases, namely a significant contribution of the intracellular pathway to total water transport and a reduction in both endfoot and IEG flow rates after AQP4 deletion.

## Discussion

We have shown how parallel extracellular and intracellular pathways established by astrocyte networks may facilitate bulk fluid flow between adjacent arterial and venous paravascular spaces. The unintuitive changes in tracer distribution upon AQP4 deletion observed *in vivo* can be explained by the inhibition of the connection between these parallel pathways.

The high polarization of AQP4 on astrocyte endfeet may distract from the presence of these water channels on the remainder of the cell. The entire astrocyte plasma membrane including all processes constitutes an exchange surface that is approximately 400 times larger than the endfoot membrane, allowing for substantial water exchange between intra- and extracellular spaces even though the AQP4 density is ten times lower there than on the endfoot^27^. The importance of such water exchange derives from the fact that a) the resistance of the ECS to water flow is higher than that of the intracellular space (Table 1), and b) two thirds of the fluid exiting the arterial PVS enter the extracellular space (Fig. 3). To follow the lower resistance intracellular route, water has to first pass from the ECS into the cells, which is facilitated by AQP4 expressed on the entire astrocyte plasma membrane.

The significance of this continuous water exchange within the tissue is evidenced when astrocytes in the central AUs are progressively replaced by generic cells that do not express AQP4 (Fig. 4). In the situation with only two perivascular AUs (i.e. omitting the contribution of the central astrocytes), the total flow rate is cut to 46%, clearly demonstrating the function of the astrocytes in reducing the effective resistance of brain tissue to water flow and facilitating fluid transit between adjacent PVS.

The water exchange between the parallel intra- and extracellular routes explains observations by Iliff *et al.* who report that AQP4 knock-out mice had a lower rate of tracer penetration from the PVS into the brain tissue than their wild type counterparts^7^. This implies reduced flow rate through IEG upon AQP4 deletion, since tracers cannot pass through AQP4. If intra- and extracellular routes served as disconnected parallel fluid pathways, deletion of AQP4 would not reduce tracer influx from the PVS through the IEG, and the flow rate through IEG would be 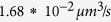 (dashed line in Fig. 3) irrespective of the presence or absence of AQP4. In contrast, deletion of AQP4 in the complete network of astrocytes reduces the flow rate through IEG (Fig. 5a). This is in line with the experimental observations.

The tracer distribution observed *in vivo* does not only depend on fluid flow through PVS and brain tissue, but also on diffusion. To assess the relative contributions of diffusion and advection to tracer movement, we consider the Péclet number^28^,


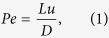


where L and u are characteristic length and velocity, respectively, and D is the diffusion coefficient of the tracer. When this dimensionless number is larger than one, advection (i.e. transport of the tracer with the flow) is more important than diffusion.

Indirect measurements of ISF velocity via tracers have yielded a range of values from 0 to 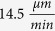
19,20. For the calculation of Pe we consider a range of 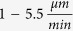, where the lower limit is the maximum for grey matter given by Rosenberg *et al.* (Fig. 4 in^19^), and the upper limit the minimum given by^20^,^29^,^30^ for the entire brain. With this, Pe in the parenchymal ECS is in the range of 0.005 to 0.275, while in the PVS it is 0.45 to 25 (see Supplementary Information S2). This means that while diffusion dominates tracer distribution in the parenchyma, supply of the tracer through the arterial and removal through the venous PVS depend on both advection and diffusion.

More relevant than the influx of artificial tracers is the fate of natural metabolites in the ISF. These are hypothesized to be cleared through the venous PVS^7^. For the baseline astrocyte network with functioning AQP4, the ratio of solute transport capacities between PVS and tissue is in the range of 0.045 to 0.61 for naturally occurring solutes (see Supplementary Information S2). This means that, under normal conditions, the solute transport capacity of the tissue (dominated by diffusion) is higher than that of the paravascular space (dependent on both advection and diffusion). Thus, the PVS appears to be the limiting segment in the solute transport pathway. In absence of AQP4, the solute transport capacity of the PVS is further diminished because the fluid flow rate through it is reduced (Fig. 5a). Consequently, even though metabolites are transported largely by diffusion in the brain tissue, their removal is nevertheless dependent on bulk flow through the PVS, and in turn on the net flow of water through both intra- and extracellular routes in the tissue. This also explains *in vivo* experimental observations of reduced solute clearance from the tissue in AQP4 knock-out mice^7^.

There are indications that the fluid flow rate through the PVS is increased during sleep^31^, presumably due to reduced resistance in the ECS as a consequence of an increase in volume of this space^31^. In our model, the reported 60% increase in ECS volume^31^ translates to a 46% reduction of ECS resistance. Even though increased ECS volume necessitates a decrease in intracellular volume and thus an increase in the corresponding water pathway’s resistance (by 25%), the overall resistance between arterial and venous PVS drops, leading to a 36% increase in total flow rate. This in turn raises the ratio of solute transport capacity in the PVS to that in the tissue to a range of 0.048 to 0.77 for naturally occurring solutes (see Supplementary Information S2). This means that during sleep, the clearance rates of larger metabolites such as amyloid beta are closer to their limit set by diffusion in the brain tissue.

Para-arterial water inflow and water secretion from capillaries into the parenchyma may coexist. Under the here investigated conditions, the observations of reduced tracer influx into the ISF in animals lacking AQP4 are reproduced as long as the arterial PVS is the main source of water influx into the parenchyma. Under nominal conditions (Fig. 8), this is even the case for ratios of capillary secretion to inflow from PVS of up to 4:1. At higher ratios, water is drained through the arterial PVS rather than supplied, and AQP4 deletion increases drainage.

The relevance of this work derives from the fact that it is currently not possible to measure interstitial fluid flow directly *in vivo*, but that flow rates have to be inferred from the evolution of tracer distribution patterns. This also implies that direct quantitative experimental validation of our model will be extremely challenging. However, our results are in agreement with qualitative experimental observations:

We show that deletion of AQP4 channels results in a reduction of IEG flow rate and increase in the flow rate through GJs, both of which have also been observed *in vivo* in AQP4 knock-out rodents^7^,^26^. Our parametric variations of the number of astrocytes and AQP4 distribution provide a rationale for the makeup of the fluid pathway structures, demonstrating that both AQP4 polarization on the astrocyte endfeet and a dense astrocyte network are required for optimal water transport between paravascular spaces.

The flow distribution between intra- and extracellular pathways is determined by the resistances of the individual routes. Inadequate choices of resistance values could yield incorrect results. To reduce this possibility, we performed a sensitivity analysis as documented in Supplementary Information S3. This analysis shows that our model is robust with respect to variation of parameters within realistic bounds.

Our model considers a pressure gradient between the arterial and venous PVS as the driving force behind the observed fluid flow. This gradient can be viewed as the result of the superposition of all driving forces acting on water on its way from arterial to venous PVS, including possible hydrostatic pressure caused by arterial wall pulsation^32^,^33^. Since the magnitude of the gradient is unknown, we prescribe a value that yields a baseline extracellular fluid flow velocity in line with experimental estimates in grey matter^19^. As a consequence, the absolute values of the here reported flow rates should also be seen as estimates. However, as discussed in Supplementary Information S3, the conclusions of the investigations are not affected by reasonable changes of the gradient magnitude.

More important than its value is the question whether such a gradient exists in the first place. The reduction of the rate of tracer distribution and metabolite clearance in mice lacking AQP4 appears to necessitate bulk flow from arterial to venous PVS, and with it a corresponding driving force gradient. Should it be the case that AQP4 deletion has secondary effects that influence fluid flow, other explanations for the observed tracer spread would have to be considered. These include flow-induced mixing and dispersion in the PVS, as well as increased effective tracer and metabolite diffusivity in the parenchyma due to Taylor dispersion^34^. Both effects could be caused by arterial pulsation and do not necessitate bulk flow.

In summary, while diffusion is the main mode of solute transport in brain tissue, it does not suffice to explain effective metabolite clearance into the venous paravascular space observed in tracer studies *in vivo*. Other modes of transport need to be taken into account as well. We have shown with this computational study that if there is bulk flow between arterial and venous paravascular spaces, astrocyte networks may serve as low resistance pathways to water flow that enhance metabolite clearance through a parallel, extracellular route. Observations of reduced metabolite clearance in animals lacking AQP4 may be explained by diminished bulk flow caused by inhibition of the interconnection between the astrocyte networks and the extracellular route.

## Additional Information

**How to cite this article**: Asgari, M. *et al.* How astrocyte networks may contribute to cerebral metabolite clearance. *Sci. Rep.* **5**, 15024; doi: 10.1038/srep15024 (2015).

## Supplementary Material

Supplementary Information

## Figures and Tables

**Table 1:** Definitions and nominal values of parameters used in the model.

Parameter	Equation	Nominal value
Pressure gradient between arterial and venous PVS	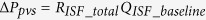	226 Pa
Capillary basement membrane resistance for half the length of one AU	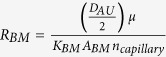	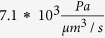
ECS resistance for half the length of one AU	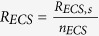	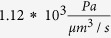
IEG resistance for the thickness of an endfoot	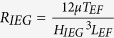	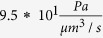
Resistance of intracellular pathway in the astrocyte processes for half the length of one AU	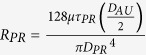	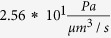
Endfoot membrane resistance	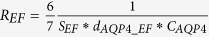	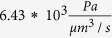
Resistance of the plasma membrane in half of the astrocyte unit	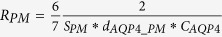	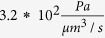
Gap junction resistance	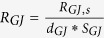	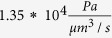

Derivations are documented in Supplementary Information S1. 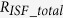 Total flow resistance in the ISF space between arterial and venous PVS; 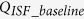 Nominal Interstitial fluid flow rate; 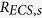 Resistance of a single ECS pathway for half the length of one AU; *D*_*AU*_ Diameter of the astrocyte unit; *n*_*capillary*_ Average number of capillaries in one AU; *K*_*BM*_ hydraulic permeability of the capillary basement membrane; *A*_*BM*_ the cross section area of basement membrane; *n*_*ECS*_ Number of parallel ECS pathways in one AU; *μ* dynamic viscosity of ISF; *T*_*EF*_ endfoot thickness; *L*_*EF*_ endfoot length; *H*_*IEG*_ thickness of IEG; *τ*_*PR*_ Tortuosity of the astrocyte processes; *D*_*PR*_ Diameter of astrocyte process; *C*_*AQP*4_ Water conductivity of a single AQP4; *S*_*EF*_ and *S*_*PM*_ Endfoot and plasma membrane surface areas, respectively; 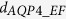 and 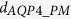 AQP4 densities over the endfoot and plasma membrane, respectively; 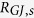 Water resistance of single gap junction channel; *d*_*GJ*_ density of gap junction channels; *S*_*GJ*_ surface area of the contact region between neighbouring astrocytes.

**Table 2:** Parameters used for the derivation of the model parameters in Table 1.

Parameter	Equation and nominal value	Ref.
**Pressures gradient**		
ISF baseline velocity in the brain grey matter	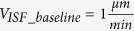	19
Range of reported ISF velocities	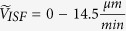	19,20
**Vascular dimensions**		
Distance between arteriole-venule pair	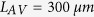	10
Capillary diameter	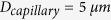	35
Capillary volume fraction in the brain	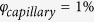	36
Cortical penetrating arteriole diameter	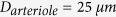	36
**Astrocyte dimensions**		
AU diameter	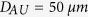	10
AU volume	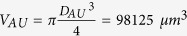	
Astrocyte soma diameter	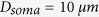	10
Surface area of astrocyte soma	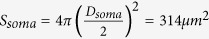	
PM to soma surface ratio	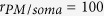	10
PM surface area	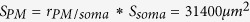	
Astrocyte process diameter	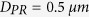	37
Process intracellular space tortuosity assuming cylindrical structures	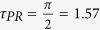	
**AQP4**		
Single AQP4 channel water conductivity	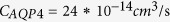	38
AQP4 density on EF	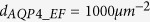	27
AQP4 density on PM (except EF)	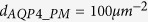	39
EF length (estimated from the fact that EF fully wraps around capillaries)	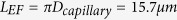	17
EF width	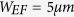	17
EF surface area	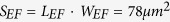	
EF thickness	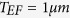	17
**GJ**		
Hydraulic diameter of a GJ channel	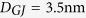	18
PM thickness	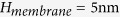	40
Resistance of single GJ channel to water flow	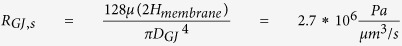	
GJ channel density in the contact region between neighbouring astrocytes	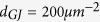	41
Surface area of the contact region between neighbouring astrocytes	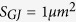	41
**ECS**		
ECS thickness	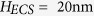	42
Inter-endfeet gap thickness	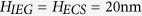	7,43
ECS volume fraction	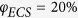	5
Width of ECS channel surrounding typical cellular features (processes)	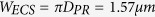	
ECS tortuosity assuming cylindrical structures	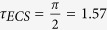	
Resistance of a single ECS pathway for half the length of one AU	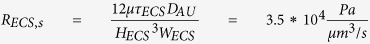	
Volume of a single ECS pathway for the length of one AU	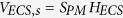	
Total ECS volume in one AU	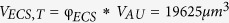	
Number of parallel ECS pathways in one AU	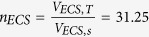	
**PVS**		
Length of PVS segment considered for a penetrating vessel	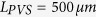	36
Thickness of PVS channel	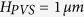	44
Hydraulic permeability of PVS	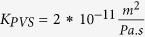	45
**Capillary BM**		
BM thickness	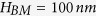	46
Hydraulic permeability of BM	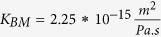	47
Volume of single capillary with the length of one AU	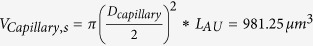	
Number of capillaries in one AU	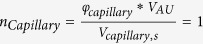	
Cross section area of BM layer	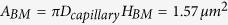	
**Diffusion**		
Diffusion coefficient for natural solutes and tracers in the brain parenchyma	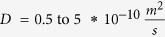	5
**Fluid properties**		
Dynamic viscosity	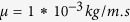	
Density	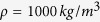	

The most prominent spatial dimensions are illustrated in the Supplementary Fig. S1. Derivations are all documented in details in Supplementary Information S1.
