# Supplementary material for: How astrocyte networks may contribute to cerebral metabolite clearance
Source: Sci Rep. 2015 Oct 14;5:15024. doi: 10.1038/srep15024 (PMC4604494; doi:10.1038/srep15024)
Supplement: Supplementary Information [file srep15024-s1.pdf]

Title of the manuscript:

How astrocyte networks may contribute to cerebral metabolite clearance

Authors:

Mahdi Asgari (MSc), Diane de Zélicourt (PhD), Vartan Kurtcuoglu (PhD)

S.1 Supplementary Information – Estimation of flow resistances

S.1.1 Parameters

Table 2 in the main text lists all parameters used, accompanied by the corresponding reference or associated equation for derived quantities. For ease of understanding, the most prominent spatial dimensions used to obtain the model parameters are illustrated in the Supplementary Fig. S1.

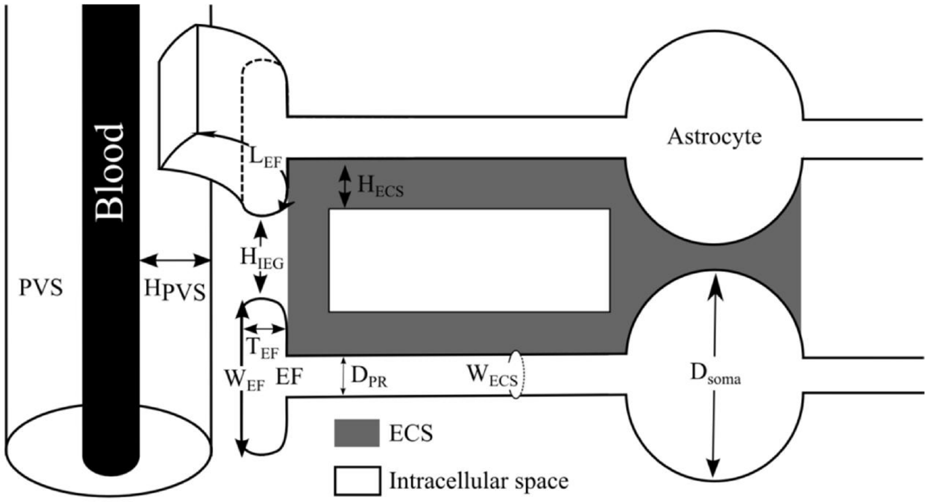

Supplementary Figure S1: Dimensions used to obtain the model parameters. Nomenclature and values of these dimensions are given in Table 2.

S.1.2 Pressure gradient between arterial and venous paravascular spaces

A pressure difference of 226 Pa (1.7 mmHg) between arterial and venous PVS is required to yield the baseline interstitial fluid flow velocity of $V_{ISF\_baseline} = 1\frac{\mu m}{min}$ (see Table 2). This pressure difference should be seen as the net value produced by the superposition of all relevant hydrostatic and osmotic pressure sources, including arterial wall pulsations[1, 2]. The stated 1.7 mmHg appear reasonable compared to the 17 mmHg blood pressure drop from arteriole to venule[3].

S.1.3 Estimation of the bulk flow resistance in channel structures of the brain micro-environment including extracellular and intra-cellular pathways.

S.1.3.1 Simplifications

Laminar flow is assumed in the ECS, which is supported by the low Reynolds number in that space,

$$Re = \frac{\rho u D_h}{\mu} < 5 * 10^{-8}, \tag{1}$$

where u is the expected flow velocity in the range of $5.5 - 14.5\ \frac{\mu m}{min}$ [4], D_h is the hydraulic diameter of the fluid path (taken to be equal to H_{ECS}), ρ and μ are, respectively, density and dynamic viscosity of the interstitial fluid.

The ECS can be viewed as a tortuous pathway that winds around cellular elements in the tissue. This may have two effects: first, tortuosity increases the effective length of a channel compared to a straight one, which is typically accounted for by correcting the straight length by a tortuosity coefficient; second, local curvatures may induce secondary flow structures that would dissipate energy and increase the effective resistance of the path. However, this second effect can be neglected herein, as demonstrated by the very low Dean number (Dn),

$$\boldsymbol{Dn = \frac{\rho u H_{ECS}}{\mu}\left(\frac{H_{ECS}}{R}\right)^{1/2} < 10^{-2}}, \tag{2}$$

where R is the characteristic radius of curvature of the pathway, which we considered to be equal to half of the cell process diameter (D_{PR}). Hence, we may retain the channel flow equations to derive the resistances of the different flow pathways, solely correcting them for the increase in effective path length.

S.1.3.2 Derivation of R_{ECS}

With the above simplifications, the resistance of one ECS pathway, $R_{ECS,s}$, can be obtained from the analytical expression for a straight channel[5],

$$\boldsymbol{R_{ECS,s}} = \frac{12\mu\tau_{ECS}(\frac{D_{AU}}{2})}{H_{ECS}{}^{3}W_{ECS}}, \tag{3}$$

where τ_{ECS} is the ECS channel tortuosity, with the product $\tau_{ECS}(\frac{D_{AU}}{2})$ representing the effective length of an ECS channel spanning half of one AU. The resistance value for one ECS pathway obtained using Equation (3) corresponds to a single route through the ECS. In practice, there are multiple ECS routes within a single astrocyte unit (AU). Using the ECS volume fraction φ_{ECS} and characteristic dimensions reported in Table 2, we can determine the volume of characteristic single ECS route, $V_{ECS,s}$, and deduce the total number of routes, n_{ECS}, required to match the total ECS volume, $V_{ECS,T}$, from the ratio of the two volumes

$$n_{ECS} = \frac{V_{ECS,T}}{V_{ECS,s}}. \tag{4}$$

Assuming that these individual ECS routes carry water in parallel, the overall ECS resistance of half the length of one AU can be approximated as:

$$R_{ECS} = \frac{R_{ECS,s}}{n_{ECS}}. \tag{5}$$

S.1.3.3 Estimation of the inter-endfeet gap resistance, R_{IEG}

Applying the analytical expression for the resistance of a straight channel (Equation (3)), and assuming the length, height and width of the IEG to be given by the endfoot thickness, T_{EF}, ECS channel thickness, H_{ECS}, and characteristic length of the endfoot contact area, L_{EF}, we obtain:

$$R_{IEG} = \frac{12\mu T_{EF}}{{H_{ECS}}^3 L_{EF}}. \tag{6}$$

Tortuosity is assumed to be 1, since the IEG is very short. Based on the observation that astrocyte endfeet can completely enwrap individual capillaries [6], the characteristic endfoot length is given the same value as the circumference of a capillary,

$$\boldsymbol{L_{EF} = \pi\, D_{capillary}}. \tag{7}$$

S.1.3.4 Estimation of the capillary basement membrane resistance, R_{BM}

To calculate the overall resistance of the capillary basement membrane layers in one AU, we need an approximation of the number of capillaries available in the astrocyte domain volume, $n_{capillary}$. To this end, we consider the capillary volume fraction $\varphi_{capillary}$ in brain tissue:

$$n_{capillary} = \frac{\varphi_{capillary} * V_{AU}}{V_{capillary,s}}, \tag{8}$$

where V_{AU} is the astrocyte domain volume and $V_{capillary,s}$ the characteristic volume of a single capillary segment of the length of one AU. $n_{capillary}$ evaluates to approximately 1, which means that the overall capillary segment length in an AU sized volume of brain tissue has the length of one AU. When we state in the description of our model that each AU is associated with one capillary, we refer to this situation.

The capillary basement membrane is rather dense. For half the length of one AU, its resistance can be calculated as

$$R_{BM} = \frac{\frac{D_{AU}}{2}\ \mu}{K_{BM}\ A_{BM}\ n_{capillary}}, \tag{9}$$

where K_{BM} is the permeability of the capillary basement membrane and A_{BM} is its cross-sectional area of this membrane calculated based on the characteristic dimensions reported in Table 2.

S.1.3.5 Estimation of intra-cellular resistances, R_{PR}

Following a similar approach, the intracellular resistance of an astrocyte process spanning half the length of one AU is obtained from resistance of cylindrical pipe of diameter, D_{PR}, and effective length $\tau_{PR}(\frac{D_{AU}}{2})$:

$$\boldsymbol{R_{PR}} = \frac{\boldsymbol{128\mu\tau_{PR}(\frac{D_{AU}}{2})}}{\boldsymbol{\pi D_{PR}}^{4}} = \boldsymbol{2.56 * 10^{1}\ \frac{Pa}{\mu m^{3}/_{s}}}. \tag{10}$$

The above equation assumes free fluid flow through the entire intra-cellular space. In practice, the volume fraction of the cytosol of retinal glial cells is > 50% [7]. Inferring a similar distribution for astrocytes would bring the intracellular pathway resistance up to $6.45 * 10^{1}\ \frac{Pa}{\mu m^{3}/_{s}}$. However, as demonstrated in our sensitivity analysis (Supplementary Information S3), this has hardly any impact on the reported results due to the dominant effect of the plasma and endfoot membrane resistances.

S.1.4 Estimations of the cell membrane resistances

S.1.4.1 Contribution of AQP4 channels to the membrane resistance

In our model, we divide the plasma membrane resistance into two components, each representing the plasma membrane in half of the astrocyte unit. Knowing the conductivity of a single AQP4 channel, C_{AQP4}, to be $24*10^{-14}$ cm^3/s [8], the contribution of AQP4 to the plasma membrane resistance is given by

$$R_{PM\_AQ} = \frac{1}{d_{AQP4\_PM}\left(\frac{S_{PM}}{2}\right) C_{AQP4}}, \tag{11}$$

where d_{AQP4_PM} is the density of AQP4 over the plasma membrane (not including the endfoot) and $S_{PM}/2$ is the surface area of the plasma membrane in half of an AU. The AQP4 contribution to the resistance of the endfoot plasma membrane, R_{EF_AQ}, is obtained according to equation (11) as well, using the density of AQP4 on the endfoot, d_{AQP4_PM}, and estimating the endfoot surface area as

$$S_{EF} = L_{EF}\, W_{EF} = \left(\pi D_{capillary}\right) D_{capillary}. \qquad (12)$$

This estimation makes use of the observation that astrocyte endfeet can completely enwrap individual capillaries with approximately the same width as the capillary diameter [6].

S.1.4.2 Overall membrane resistance and its change after AQP4 deletion

The overall resistance of the astrocyte plasma membrane in one astrocyte unit is given by

$$R_{PM} = \frac{R_{PM\_AQ} R_{PM\_mb}}{R_{PM\_AQ} + R_{PM\_mb}}, \qquad (13)$$

where R_{PM_AQ} is the resistance of AQP4 channels on the plasma membrane obtained from equation (11) and R_{PM_mb} is the resistance of the membrane itself. While the contribution of the AQP4 channels was estimated above, that of the membrane still needs to be determined. We make use of the fact that after AQP4 deletion, the overall resistance $R_{PM_{AQ-/-}}$ is seven times higher than the resistance in the presence of AQP4 channels [9], R_{PM}. Together with equation (13), this yields the following relationships:

$$\boldsymbol{R_{PM_{AQ-/-}} = R_{PM\_mb}}, \qquad (14)$$

$$\boldsymbol{R_{PM_{AQ-/-}} = 7R_{PM}}, \qquad (15)$$

from which we can deduce

$$\boldsymbol{R_{PM} = \frac{6}{7} R_{PM\_AQ}}. \qquad (16)$$

The overall resistance of the endfoot membrane, R_{EF}, can be derived similarly.

S.1.5 Gap junction resistance

The typical hydraulic diameter of a gap junction channel, D_{GJ}, is 2.5-4.5 nm [10]. As GJ channels connect two plasma membranes of 4-6 nm thickness, $H_{membrane}$ [11], their length is approximately in the range of 8-12 nm. Navier-Stokes equations are principally only valid for continuum fluids. Nevertheless, they have been shown to provide good estimates for water dynamics in nanochannels [12], with diameters as small as 5-10 times the width of an individual fluid molecule's diameter [13]. Accordingly, we estimate the resistance of single GJ channel based on the assumption of Hagen-Poiseuille flow through a circular cylindrical channel with diameter D_{GJ} and length $2H_{membrane}$:

$$\boldsymbol{R_{GJ,s}} = \frac{\boldsymbol{128\mu(2H_{membrane})}}{\boldsymbol{\pi D_{GJ}}^4}. \tag{17}$$

The total resistance of a gap junction is obtained by considering the density of GJ channels d_{GJ} of $200\ \mu m^{-2}$ over a contact area S_{GJ} of $1\ \mu m^2$ estimated from the figures in the reference [14]:

$$R_{GJ} = \frac{R_{GJ,s}}{d_{GJ} * S_{GJ}}. \tag{18}$$

S.1.6 Water secretion by capillaries

To assess the effect of possible water secretion by capillaries[15], we introduce an expanded version of the model described in the manuscript. This is shown below in Supplementary Fig. S2. Water secretion by capillaries is enforced by flow sources (or current sources in the electric analogue). There is one source per AU length of capillary, thus 6 sources with the same secretion rate in total. The secretion rate and the pressure drop from arterial to venous PVS are set such that the baseline parenchyma flow rate (combination of the total influx through arterial PVS, including IEG, EF and BM, and secretion by capillaries) is always maintained. Thus when the secretion rate is step-wise increased from its nominal value of zero, the pressure drop from arterial to venous PVS is decreased to keep the baseline parenchyma flow rate (Supplementary Table S1). Even when capillary secretion accounts for the entire

parenchymal water influx, a pressure gradient from arterial and venous PVS is still necessary to ensure the prescribed ISF flow velocity. In absence of this gradient, there would be outflow through both arterial and venous PVS, yielding zero net ISF velocity.

When the effect of AQP4 deletion is studied, the secretion rate is reduced by 31%[16] without further adjustment of the above-determined inter-PVS pressure drop (Supplementary Table S1). This 31% reduction is based on observations in glial-conditional AQP4 knockout mice after systemic hypoosmotic stress[16]. Under normal osmotic conditions, one can expect less reduction in the secretion rate upon AQP4 deletion, since water secretion is an active process that can be upregulated. We investigate the effects of both higher and lower rates of secretion reduction in the sensitivity analysis in Supplementary Information S3.

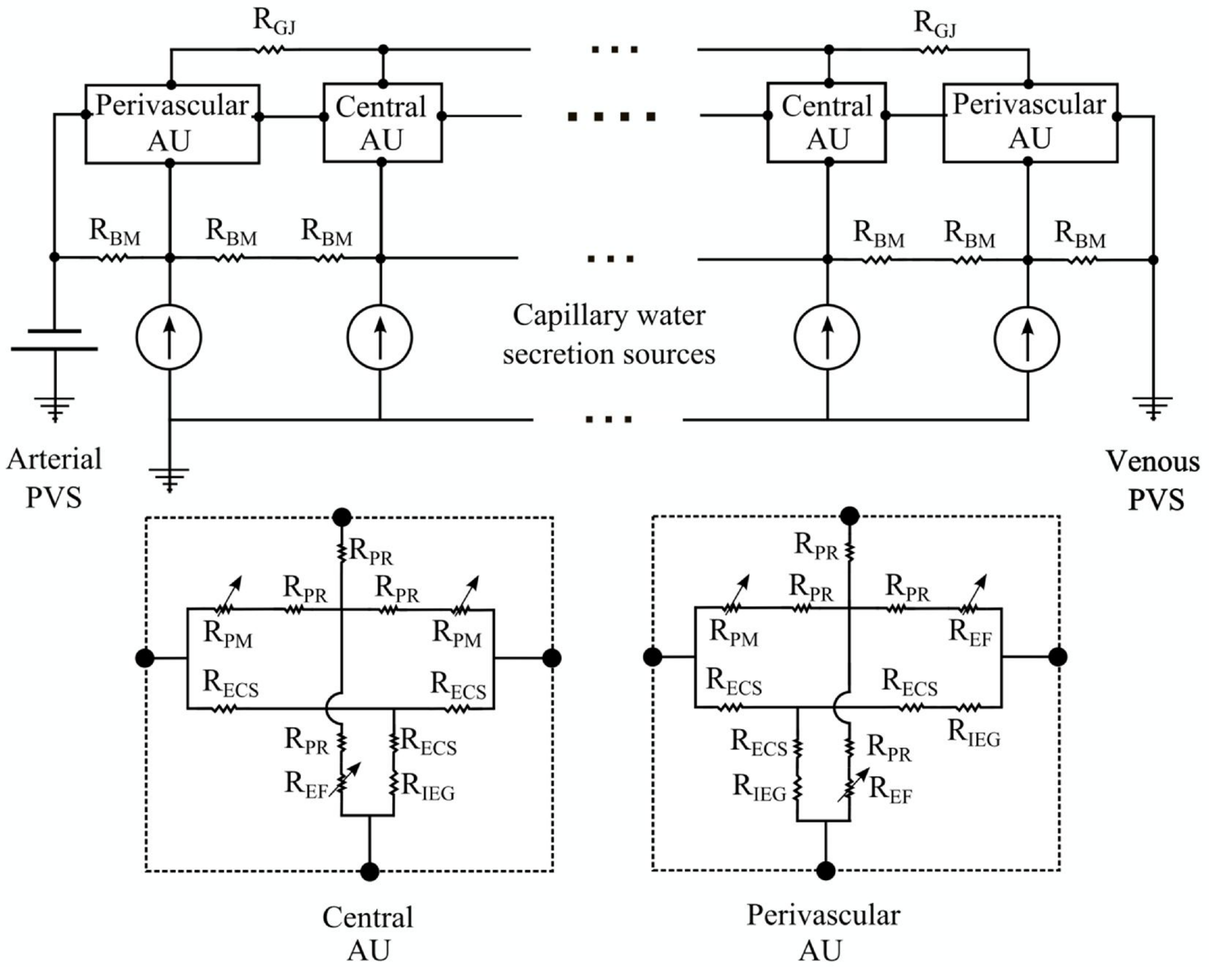

Supplementary Figure S2: Electrical analogue model of cerebral water transport between arterial and venous paravascular spaces (PVS) including water secretion sources at the capillary level in addition to the elements described in Figure 2 (main text). Definitions of the abbreviations referring to the physical model domain are given in Figure 1 (main text). Arterial and venous paravascular spaces are connected by resistances (R) representing the resistance to fluid flow of capillary basement membrane (BM) segments and astrocyte units (AU). Each astrocyte unit (AU) includes resistances of both intracellular (cell processes, PR) and extracellular (ECS) pathways which are linked by membrane resistances, namely those of the astrocyte endfoot membrane (EF) and the remainder of the astrocyte plasma membrane (PM). Since these membrane resistances are dependent on the AQP4 expression level, they are indicated as variable resistances (arrows). Gap junction (GJ) resistances connect the intracellular spaces of two neighbouring astrocytes.

Supplementary Table S1: Rates of water secretion from capillaries in normal and AQP4 knock-out cases for different levels of contribution of secretion to overall water influx. The corresponding pressure drop between arterial and venous paravascular spaces is also reported.

Capillary secretion rate as fraction of overall water inflow rate [%]	Inter-PVSs pressure drop [Pa]	Capillary secretion rate in normal case $[10^{-2}\ \frac{\mu m^3}{s}]$	Capillary secretion rate in AQP4 knock-out case $[\ 10^{-2}\ \frac{\mu m^3}{s}]$
0	226	0	0
20%	202.98	0.91	0.63
40%	180.09	1.82	1.26
60%	157.71	2.72	1.88
80%	134.32	3.63	2.5
100%	111.94	4.54	3.13

S.2 Supplementary Information – Solute transport analysis

S.2.1 Relative role of diffusion and advection in solute transport in the tissue and PVS

To evaluate the relative contribution of advection and diffusion to solute transport in the ECS, we consider the non-dimensional Péclet number:

$$Pe = \frac{Lu}{D}, \qquad (19)$$

where L is the characteristic length, u is the flow velocity and D is the diffusion coefficient of the solute. For naturally occurring solutes and tracers commonly used to study ISF flow in the brain, D ranges between $5*10^{-11}$ and $5*10^{-10}$ m^2/s [17]. As these solutes are either produced throughout the tissue or injected at a given location in the tissue, we set the diffusion length to half the distance between arterial and venous PVS, namely $L_{AV}/_2$. For the ISF flow velocity, there is a range of reported values from $0 - 1\ \frac{\mu m}{min}$ specifically for grey matter[18] to brain averaged values of $5.5 - 14.5\ \frac{\mu m}{min}$ [4]. For the calculation of Pe we consider a range of $1 - 5.5 \frac{\mu m}{min}$, where the lower limit is the maximum for grey matter given by Rosenberg et al (Fig. 4 in [18]), and the upper limit the minimum given by [4, 19, 20] for the entire brain. This

yields Pe in the range of 0.005 to 0.275 in the ECS. Solute transport in the ECS is thus dominated by diffusion.

For the corresponding analysis in the PVS, we need to determine the bulk flow velocity therein based on the integrated value of water transfer between PVS and parenchyma. We consider a PVS segment of length corresponding to that of a penetrating arteriole in the rodent cortex, $L_{PVS} = 500\mu m$ [21]. Considering the flow rate from PVS to tissue through a single astrocyte endfoot and its neighbouring inter-endfeet-gap and integrating this value over the length of considered paravascular space using the characteristic dimensions reported in Table 2, we obtain

$$u_{PVS} = \frac{L_{PVS}}{(W_{EF}+H_{IEG})}\frac{\pi D_{arteriole}}{L_{EF}}\frac{Q_{unit}}{D_{arteriole}\,H_{PVS}}. \quad (20)$$

This yields fluid flow velocity between $0.9 - 5\frac{\mu m}{s}$ in the PVS velocities of 1 - 5.5 $\frac{\boldsymbol{\mu m}}{\boldsymbol{min}}$ in the ECS. Accordingly, the Péclet number ranges between 0.45 and 25 in the PVS for a diffusion length of half the PVS length, ${L_{PVS}}/{2}$. Advection must thus not be neglected in the PVS, and is the dominant factor in the transport of large solutes such as amyloid beta.

S.2.2 Solute transport capacity in ECS and PVS

We use the metrics of convective and diffusive fluxes to calculate the upper limit of solute transport through ECS and PVS, referring to this limit as solute transport capacity. Convective and diffusive fluxes are defined as:

$$J_c = uAC,$$
$$J_d = DA\frac{\partial C}{\partial x}, \quad (21)$$

where D is the solute's diffusion coefficient, A the surface area perpendicular to the desired flux direction, C the solute concentration, $\frac{\partial C}{\partial x}$ the concentration gradient in the flux direction and u the fluid velocity.

To compare the PVS and tissue transport capacities, let us consider a segment of PVS of length L_{PVS} as illustrated in Supplementary Fig. S3, and the solute fluxes in and out of that segment. Since solutes could enter the PVS segment from whole the length of it, the diffusion length in PVS is taken as ${L_{PVS}}/{2}$, then the flux of solutes through this segment of PVS is governed by advection and diffusion as follows:

$$J_{PVS} = J_{c\_PVS} + J_{d\_PVS} = u_{PVS}\, P_{PVS}\, H_{PVS}\, C + D\, P_{PVS}\, H_{PVS}\, \frac{C}{{L_{PVS}}/{2}}, \tag{22}$$

where u_{PVS} is the velocity in the PVS and P_{pvs} and H_{pvs} are the PVS circumference and thickness, respectively. Solutes (metabolites) are produced throughout the tissue and enter the PVS through IEG. Their diffusion length is thus taken as ${L_{AV}}/{2}$. The solute flux from tissue to PVS is then written as:

$$J_{ECS} = J_{c\_ECS} + J_{d\_ECS} = u_{ECS}\, r_{IEG}\, P_{PVS}\, L_{PVS}\, C + D\, r_{IEG}\, P_{PVS}\, L_{PVS}\, \frac{C}{{L_{AV}}/{2}}, \tag{23}$$

where the product $P_{PVS}L_{PVS}$ is the surface of the PVS segment under consideration and r_{IEG} the proportion of PVS surface covered by IEG which allows the movement of solutes between PVS and tissue.

The ratio of the solute transport capacity in PVS to tissue becomes:

$$\sigma = \frac{J_{PVS}}{J_{ECS}} = \frac{u_{PVS}\, P_{PVS}\, H_{PVS}\, C + D\, P_{PVS}\, H_{PVS}\, \frac{C}{{L_{PVS}}/{2}}}{u_{ECS}\, r_{IEG}\, P_{PVS}\, L_{PVS}\, C + D\, r_{IEG}\, P_{PVS}\, L_{PVS}\, \frac{C}{{L_{AV}}/{2}}}. \tag{24}$$

Since we have already discussed the Péclet number in PVS and ECS, we rewrite the above equation based on this number definition for PVS and ECS:

$$\sigma = \frac{J_{PVS}}{J_{ECS}} = \frac{u_{PVS}\, H_{PVS}\left(1 + \frac{1}{Pe_{PVS}}\right)}{\frac{2\, L_{PVS}}{L_{AV}}\, D\, r_{IEG}\, (1 + Pe_{ECS})}. \tag{25}$$

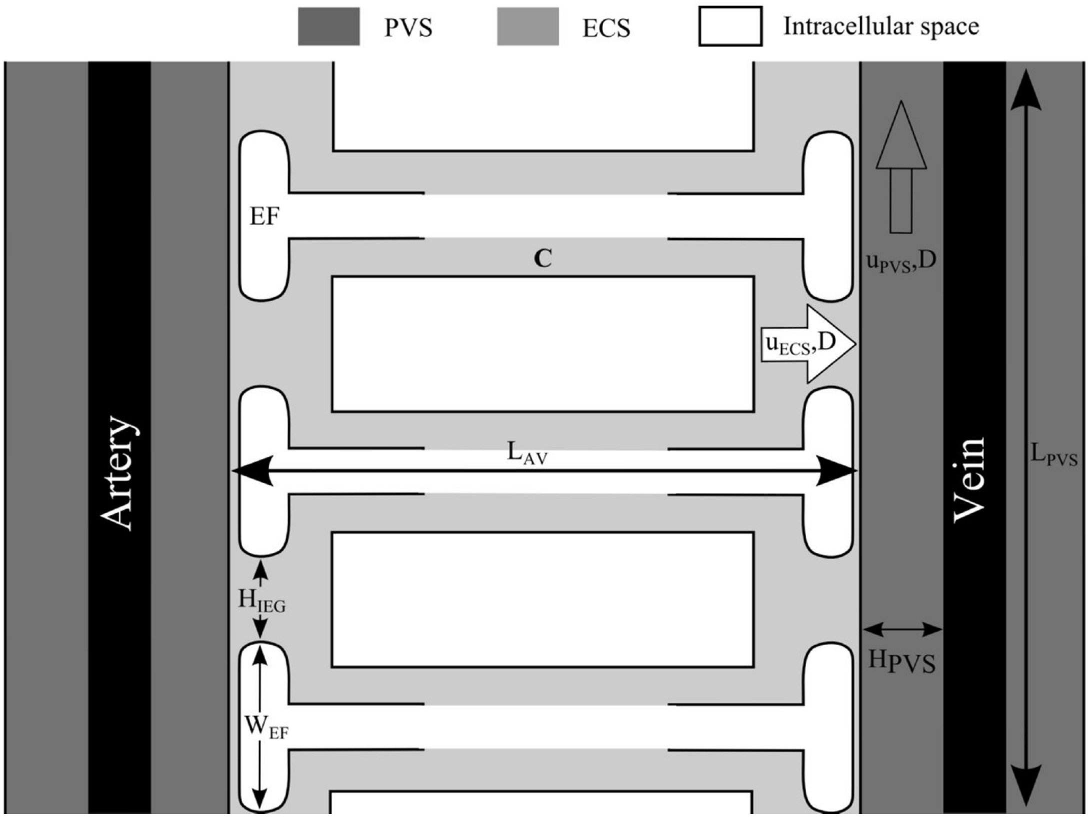

Supplementary Figure S3: Dimensions used to obtain the solute transport capacity ratio in equation (24) and (25). C represents the solute concentration in the center of the tissue.

S.2.2.1 Solute transport capacity in the awake state

Here we consider the thickness of paravascular space, H_{PVS}, to be 1 μm [22], r_{IEG}=1/25 referring to Table 2, $u_{PVS} = 0.9 - 5\ \frac{\mu m}{s}$ as estimated in S2.1, and the diffusion coefficients of natural solutes in the brain and of common tracers, D, to be in the range of $5*10^{-11} - 5*10^{-10}\frac{m^2}{s}$ [17]. With these values, theadvective to diffusive transport rate ratio ranges between:

$$\sigma = 0.045 - 0.61. \tag{26}$$

In the case of AQP4 knock-out, this ratio decreases due to the 44% reduction in overall water flow rate from PVS to tissue (see Results section):

$$\sigma_{AQP4-/-} = 0.038 - 0.39. \tag{27}$$

S.2.2.2 Solute transport capacity during sleep

It has been reported that ECS volume increases by 60% in mice during sleep. Based on a 1:4 initial ratio of ECS to intracellular space volume, this translates to a 15% reduction of intracellular volume during sleep. Under the assumption that the volume changes are caused by equal relative changes in all relevant dimensions, the ECS resistance decreases by 46% (equation (3)) while the resistance of the intracellular pathway increases by 25%. The combined effect is an increase of the total flow rate from PVS to tissue by 36% (see Supplementary Fig. S4). The solute transport capacity then ranges between 0.048 – 0.77.

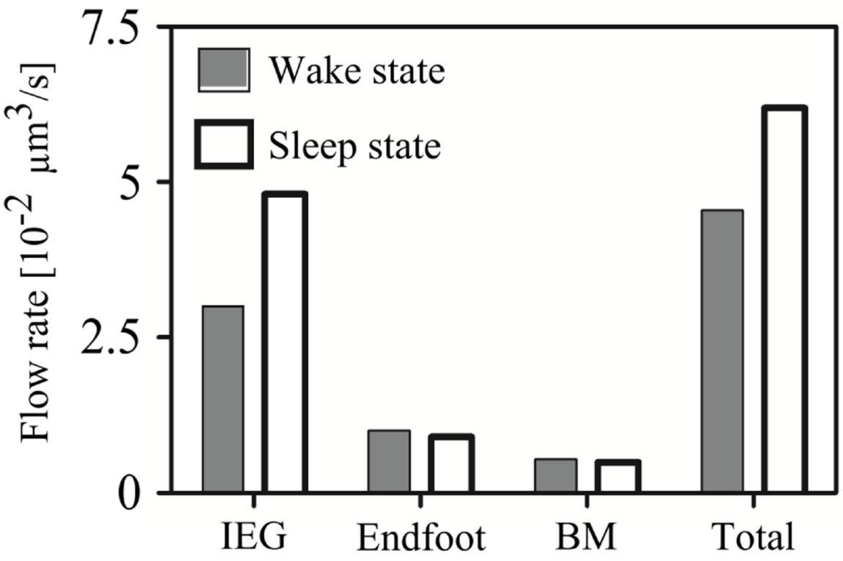

Supplementary Figure S4: Flow rates from PVS to tissue through IEG, endfoot, capillary basement membrane and in total during wake (dark bars) and sleep states (light bars).

S.3 Supplementary Information – Sensitivity analyses

We performed sensitivity analyses to ensure that the conclusions drawn in this work are not biased by the choice of model parameter values. Analyses for resistances are summarized in Supplementary Table S2, where flow rates under normal and AQP4 knock-out conditions are reported for the listed upper and lower bounds of parameters. The reported flow rates are normalized by the total flow rate under normal conditions. For the GJ resistance, the upper parameter bound was set to infinity, modelling the absence of GJs, while the lower bound was set to the endfoot membrane resistance, as this is the most water conductive part of the plasma membrane. The intracellular space is not just a fluid filled void; the volume fraction occupied by cell organelles and other solid components that offer resistance to water flow have to be taken into account. Thus, the upper bound of intracellular resistance was set based on the reported approximations of the cytosol volume fraction in glial cells (the corresponding calculations are described in Supplementary Information S1). Other resistances were varied by one order of magnitude in either direction of their respective nominal value.

Supplementary Table S3 summarizes the results of the sensitivity analyses for changes in the baseline ISF velocity based on which the pressure drop between arterial and venous PVS is derived. Since the nominal ISF velocity used in the main text is at the very lower end of the values reported in the literature, we analysed increased velocities up to $5.5\ \frac{\mu m}{min}$. We do not consider higher values to be representative of grey matter, as they are derived from measurements on the entire brain and thus include the effects of white matter as well as paravascular spaces. While absolute flow rates change, normalized values and their relative change upon AQP4 deletion are not affected. This is due to the linearity of the model for the nominal ISF velocity and inter-PVS pressure drop (in the absence of capillary secretion described in Supplementary Information S1).

Supplementary Table S4 shows the results of sensitivity analyses for different values of capillary water secretion reduction after AQP4 deletion in the expanded model described in Supplementary Information

S1. The nominal value for the secretion reduction is 31% based on experimental evidence[16]. As mentioned in the model description, this nominal value is likely too high. We have thus set the lower bound in the sensitivity analysis to 0%, i.e. no reduction in water secretion. The upper bound is set to 50%. The expected reduction of flow rate through the periarterial IEG in the full network of astrocytes after AQP4 deletion and its increase in the incomplete network are reproduced with all levels of secretion reduction as long as the PVS is responsible for at least 60% of the water supply to the parenchyma.

The conclusions drawn in this work are that a) flow rate through IEG is reduced upon AQP4 deletion in a complete astrocyte network and b) that the flow rate is not reduced in an incomplete network without central astrocytes when AQP4 is deleted. The sensitivity analyses reported in Supplementary Tables S2, S3 and S4 show that both observations hold for all considered parameter variations as long as the PVS is the main source of water flux into the parenchyma.

Supplementary Table S2: Sensitivity of the results to changes in resistance values. Results for both full and incomplete networks are provided as flow rates normalized by the corresponding total flow rate under normal conditions.

Resistance		Value $[\frac{Pa}{\mu m^3/s}]$	Model condition: Normal or AQP4-/-	Normalized flow rates							
				Full network of astrocytes (6AU)				Incomplete network (2 perivascular AU)			
				IEG	Endfoot	BM	Total	IEG	Endfoot	BM	Total
R_{ECS}<br>R_{PM}<br>R_{EF}<br>R_{GJ}<br>R_{PR}<br>R_{IEG}<br>R_{BM}	Baseline resistance values	$1.12 * 10^3$<br>$3.2 * 10^2$<br>$6.43 * 10^3$<br>$1.35 * 10^4$<br>$2.56 * 10^1$<br>$9.5 * 10^1$<br>$7.1 * 10^3$	Normal<br>AQP4-/-	0.66<br>0.46	0.22<br>0.024	0.11<br>0.08	1<br>0.57	0.67<br>0.76	0.21<br>0.038	0.12<br>0.13	1<br>0.93
R_{ECS}	lower bound	$1.12 * 10^2$	Normal	0.93	0.043	0.27	1	0.93	0.043	0.03	1
			AQP4-/-	0.83	0.006	0.02	0.87	0.95	0.007	0.03	0.983
	upper bound	$1.12 * 10^4$	Normal	0.17	0.53	0.3	1	0.17	0.04	0.04	1
			AQP4-/-	0.13	0.068	0.18	0.37	0.28	0.011	0.05	0.90
R_{PM}<br>R_{EF}	lower bound	$3.2 * 10^1$<br>$6.43 * 10^2$	Normal	0.25	0.69	0.05	1	0.25	0.69	0.06	1
			AQP4-/-	0.2	0.095	0.04	0.33	0.5	0.23	0.1	0.85
	upper bound	$3.2 * 10^3$<br>$6.43 * 10^4$	Normal	0.82	0.03	0.14	1	0.82	0.026	0.14	1
			AQP4-/-	0.73	0.005	0.12	0.86	0.84	0.004	0.14	0.99
R_{GJ}	lower bound	$6.43 * 10^3$	Normal	0.65	0.22	0.12	1				
			AQP4-/-	0.47	0.026	0.08	0.58				
	upper bound	$\rightarrow \infty$	Normal	0.67	0.22	0.12	1				
			AQP4-/-	0.45	0.022	0.08	0.54				
R_{PR}	upper bound	$6.45 * 10^1$	Normal	0.66	0.22	0.12	1	0.67	0.21	0.12	1
			AQP4-/-	0.48	0.023	0.08	0.59	0.76	0.04	0.13	0.93
R_{IEG}	lower bound	9.5	Normal	0.67	0.21	0.11	1	0.68	0.2	0.12	1
			AQP4-/-	0.48	0.021	0.07	0.56	0.78	0.038	0.13	0.94
	upper bound	$9.5 * 10^2$	Normal	0.57	0.25	0.16	1	0.57	0.26	0.17	1
			AQP4-/-	0.42	0.03	0.12	0.575	0.68	0.04	0.18	0.9
R_{BM}	lower bound	$7.1 * 10^2$	Normal	0.35	0.13	0.52	1	0.32	0.1	0.58	1
			AQP4-/-	0.27	0.013	0.41	0.7	0.35	0.01	0.59	0.96
	upper bound	$7.1 * 10^4$	Normal	0.74	0.24	0.01	1	0.75	0.24	0.01	1
			AQP4-/-	0.51	0.026	0.01	0.54	0.86	0.04	0.02	0.92

Supplementary Table S3: Sensitivity of the reported flow rates to changes in nominal ISF flow velocity and corresponding inter-PVS pressure drop. Results for both full and incomplete networks are provided as flow rates normalized by the corresponding total flow rate under normal conditions.

V_{ISF}	Inter-PVS Pressure drop [Pa]	**Model condition:** Normal or AQP4-/-	**Normalized flow rates**							
			Full network of astrocytes (6 AUs)				Incomplete network (2 perivascular AUs)			
			IEG	Endfoot	BM	Total	IEG	Endfoot	BM	Total
Baseline value $1\ \frac{\mu m}{min}$	226	Normal	0.66	0.22	0.11	1	0.67	0.21	0.12	1
		AQP4-/-	0.46	0.024	0.08	0.57	0.76	0.038	0.13	0.93
$2.5\ \frac{\mu m}{min}$	565	Normal	0.66	0.22	0.11	1	0.67	0.21	0.12	1
		AQP4-/-	0.46	0.024	0.078	0.57	0.76	0.04	0.13	0.93
$5.5\ \frac{\mu m}{min}$	1243	Normal	0.66	0.22	0.11	1	0.67	0.21	0.12	1
		AQP4-/-	0.46	0.024	0.078	0.57	0.76	0.04	0.13	0.93

Supplementary Table S4: Sensitivity of the reported IEG flow rates to changes in capillary water secretion reduction after AQP4 deletion. Results for different capillary secretion rates and for both full and incomplete networks are provided.

			**Flow rates through arterial IEG $[10^{-2}\ \frac{\mu m^3}{s}]$**											
			Full network of astrocytes (6 AUs)						Incomplete network (2 perivascular AUs)					
**Reduction in capillary water secretion after AQP4-/- [%]**		**Capillary secretion as fraction of total inflow [%]**	**0**	**20**	**40**	**60**	**80**	**100**	**0**	**20**	**40**	**60**	**80**	**100**
		**Model condition:** Normal or AQP4-/-												
	**0**	Normal	3	2.4	1.8	1.2	0.6	0	1.4	0.95	0.52	0.08	-0.36	-0.79
		AQP4-/-	2.1	1.53	0.97	0.4	-0.17	-0.73	1.6	1.08	0.57	0.05	-0.47	-0.99
Nominal value	**31**	Normal	3	2.4	1.8	1.2	0.6	0	1.4	0.95	0.52	0.08	-0.36	-0.79
		AQP4-/-	2.1	1.64	1.18	0.73	0.27	-0.18	1.6	1.19	0.78	0.38	-0.03	-0.44
	**50**	Normal	3	2.4	1.8	1.2	0.6	0	1.4	0.95	0.52	0.08	-0.36	-0.79
		AQP4-/-	2.1	1.7	1.32	0.93	0.54	0.15	1.6	1.26	0.92	0.58	0.24	-0.1

Supplementary References

1. Iliff J. J., *et al.* Cerebral arterial pulsation drives paravascular CSF-interstitial fluid exchange in the murine brain. *J Neurosci* 33, 18190-18199 (2013).

2. Rennels M. L., Gregory T. F., Blaumanis O. R., Fujimoto K. & Grady P. A. Evidence for a 'paravascular' fluid circulation in the mammalian central nervous system, provided by the rapid distribution of tracer protein throughout the brain from the subarachnoid space. *Brain Res* 326, 47-63 (1985).

3. Blaustein M. P., Kao J. P. Y., Matteson D. R. & Blaustein M. P. *Cellular physiology and neurophysiology: Mosby Physiology Monograph Series*, 2nd edn. Elsevier Health Sciences (2012).

4. Nicholson C. Diffusion and related transport mechanisms in brain tissue. *Rep Prog Phys* 64, 815-884 (2001).

5. Kirby B. J. *Micro- and nanoscale fluid mechanics : transport in microfluidic devices*. Cambridge University Press (2010).

6. Mathiisen T. M., Lehre K. P., Danbolt N. C. & Ottersen O. P. The perivascular astroglial sheath provides a complete covering of the brain microvessels: an electron microscopic 3D reconstruction. *Glia* 58, 1094-1103 (2010).

7. Reichenbach A. Organelle-free cytoplasmic volume fraction of rabbit retinal Muller (glial) cells. *J Hirnforsch* 30, 513-516 (1989).

8. Yang B. & Verkman A. S. Water and glycerol permeabilities of aquaporins 1-5 and MIP determined quantitatively by expression of epitope-tagged constructs in Xenopus oocytes. *J Biol Chem* 272, 16140-16146 (1997).

9. Solenov E., Watanabe H., Manley G. T. & Verkman A. S. Sevenfold-reduced osmotic water permeability in primary astrocyte cultures from AQP-4-deficient mice, measured by a fluorescence quenching method. *Am J Physiol Cell Physiol* 286, C426-432 (2004).

10. Weber P. A., Chang H. C., Spaeth K. E., Nitsche J. M. & Nicholson B. J. The permeability of gap junction channels to probes of different size is dependent on connexin composition and permeant-pore affinities. *Biophys J* 87, 958-973 (2004).

11. Huang C. & Thompson T. E. Properties of lipid bilayer membranes separating two aqueous phases: determination of membrane thickness. *J Mol Biol* 13, 183-193 (1965).

12. **Popadic A., Walther J. H., Koumoutsakos P. & Praprotnik M. Continuum simulations of water flow in carbon nanotube membranes. *New J Phys* 16, (2014).**

13. **Travis K. P., Todd B. D. & Evans D. J. Poiseuille flow of molecular fluids. *Physica A* 240, 315-327 (1997).**

14. **Nagy J. I. & Rash J. E. Connexins and gap junctions of astrocytes and oligodendrocytes in the CNS. *Brain Res Brain Res Rev* 32, 29-44 (2000).**

15. **Brinker T., Stopa E., Morrison J. & Klinge P. A new look at cerebrospinal fluid circulation. *Fluids Barriers CNS* 11, 10 (2014).**

16. **Haj-Yasein N. N.*, et al.* Glial-conditional deletion of aquaporin-4 (Aqp4) reduces blood-brain water uptake and confers barrier function on perivascular astrocyte endfeet. *Proc Natl Acad Sci U S A* 108, 17815-17820 (2011).**

17. **Sykova E. & Nicholson C. Diffusion in brain extracellular space. *Physiol Rev* 88, 1277-1340 (2008).**

18. **Rosenberg G. A., Kyner W. T. & Estrada E. Bulk flow of brain interstitial fluid under normal and hyperosmolar conditions. *Am J Physiol* 238, F42-49 (1980).**

19. **Cserr H. F., Cooper D. N., Suri P. K. & Patlak C. S. Efflux of radiolabeled polyethylene glycols and albumin from rat brain. *Am J Physiol* 240, F319-328 (1981).**

20. **Szentistvanyi I., Patlak C. S., Ellis R. A. & Cserr H. F. Drainage of interstitial fluid from different regions of rat brain. *Am J Physiol* 246, F835-844 (1984).**

21. **Tsai P. S.*, et al.* Correlations of neuronal and microvascular densities in murine cortex revealed by direct counting and colocalization of nuclei and vessels. *J Neurosci* 29, 14553-14570 (2009).**

22. **Ichimura T., Fraser P. A. & Cserr H. F. Distribution of extracellular tracers in perivascular spaces of the rat brain. *Brain Res* 545, 103-113 (1991).**
